# Carbon Nanomaterials for Enhancing the Thermal, Physical and Rheological Properties of Asphalt Binders

**DOI:** 10.3390/ma14102585

**Published:** 2021-05-16

**Authors:** Zhelun Li, Xin Yu, Yangshi Liang, Shaopeng Wu

**Affiliations:** State Key Laboratory of Silicate Materials for Architectures, Wuhan University of Technology, Wuhan 430070, China; lizhelun@whut.edu.cn (Z.L.); yu-xin@whut.edu.cn (X.Y.); liangyangshi@whut.edu.cn (Y.L.)

**Keywords:** graphene, carbon nanotubes, asphalt binder, thermal conductivity, physical properties, rheological properties

## Abstract

Effective thermal conduction modification in asphalt binders is beneficial to reducing pavement surface temperature and relieving the urban heat island (UHI) effect in the utilization of solar harvesting and snow melting pavements. This study investigated the performance of two nanometer-sized modifiers, graphene (Gr) and carbon nanotubes (CNTs), on enhancing the thermal, physical and rheological properties of asphalt binders. Measurements depending on a transient plant source method proved that both Gr and CNTs linearly increased the thermal conductivity and thermal diffusivity of asphalt binders, and while 5% Gr by volume of matrix asphalt contributed to 300% increments, 5% CNTs increased the two parameters of asphalt binders by nearly 72% at 20 °C. Meanwhile, a series of empirical and rheological properties experiments were conducted. The results demonstrated the temperature susceptibility reduction and high-temperature properties promotion of asphalt binders by adding Gr or CNTs. The variation trends in the anti-cracking properties of asphalt binders modified by Gr and CNTs with the modifier content differed at low temperatures, which may be due to the unique nature of Gr. In conclusion, Gr, whose optimal content is 3% by volume of matrix asphalt, provides superior application potential for solar harvesting and snow melting pavements in comparison to CNTs due to its comprehensive contributions to thermal properties, construction feasibility, high-temperature performance and low-temperature performance of asphalt binders.

## 1. Introduction

Asphalt is a kind of viscoelastic material, which is widely applied in pavement construction. It is compounded with heavy hydrocarbons and can be obtained by natural deposition or as a by-product of the crude petroleum industry [1]. The most widespread application of asphalt is as the binder material of the pavement surface [2]. However, the black color of asphalt gives rise to high pavement surface temperatures due to the strong absorption of solar radiation, especially during the summer, which plays a crucial role in the urban heat island (UHI) effect and a number of pavement diseases including thermo-oxidative aging and rutting under traffic loads [3,4]. Although several new concepts have emerged in recent years, including reflective pavements, porous pavements, evaporative pavements and water retentive pavements [5], mainly focusing on increasing the pavement surface albedo or holding water for longer periods of time, their weaknesses in terms of glare hazards, possible environment-unfriendly issues and less-durable structures have prevented large-scale applications out of the laboratory [6]. Therefore, without changing the aggregate gradation, optimizing the thermal parameters of asphalt concrete by substituting thermal conductive or insulative materials for a portion of corresponding-sized mineral powder, fine aggregates or even coarse aggregates has attracted the attention of researchers to control the temperature of asphalt pavements. 

One method that follows this principle is reducing the thermal conductivity of asphalt concrete. Du et al. [4] designed a gradient thermal conductivity system in layered asphalt pavement by incorporating three dosages of floating beads with low thermal conductivity into asphalt, proposing to reduce the pavement temperature during the day and cut down the accumulated heat that would be released back to air at night. Feng et al. [7] substituted crushed ceramic waste aggregates for a percentage of the coarse aggregates in asphalt concrete and reported the reduction of thermal conductivity and the narrowing of the pavement temperature gradient, resulting in the decrease of pavement temperature.

Another method is to employ asphalt pavement as a solar heat collector, which harvests solar energy at sunny days and carries the heat away by circulating fluid through deep embedded pipes to store for de-icing pavement or for heating inhabitant buildings at cold days [3,8,9]. From this idea the concepts of solar harvesting and snow melting pavements were derived, under a resource-economical and environment-friendly vision. To increase the energy harvesting efficiency and accelerate the heat transfer between the pavement surface and embedded pipes, researchers have been dedicated to enhancing the thermal conductivity of asphalt concrete. Dawson et al. [8] replaced limestone aggregates with quartzite in asphalt concrete, and the results showed that the full replacement could increase the thermal conductivity of asphalt concrete by about 135%. Simulations proved its potential to reduce pavement surface temperature while increasing the temperature at a 50 mm depth in the pavement, which signified the capability of the pavement heat collector system. Pan et al. [10,11] and Chen et al. [12] prepared thermal conductive asphalt concretes by replacing part of the mineral filler with graphite powders, and testified their advancements on solar harvesting efficiency as well as the feasibility of utilizing the asphalt solar collector to melt snow. Vo, H.V. and Park [13], Tang et al. [14] found that the cooperation of graphite and carbon fiber contributed to more remarkable thermal conductivity increments compared to their individual properties, after partially substituting mineral filler due to the formation of conductive networks in asphalt concrete. Furthermore, the effectiveness of various combinations of graphite, carbon black and carbon fiber for improving the thermal conductivity of solar harvesting and snow melting pavements was validated in the study of Bai et al. [15].

It has been observed that carbon materials, especially graphite, are popular options as thermal conductive additives in asphalt pavements. The reasons always concentrate on the common traits of the “carbon family” including good thermal conductivity, strong corrosion resistance, general chemical inertia, and close density with mineral filler of asphalt concrete. In this case, two well-known advance carbon nanomaterials, graphene (Gr) and carbon nanotubes (CNTs), deserve our anticipation for their application in solar harvesting and snow melting pavements. Gr is a two-dimensional single layer of carbon atoms arranged in a hexagonal lattice structure and strongly connected by sp^2^ hybridized orbitals C=C double bonds [16]. CNTs can be described as rolling one or more graphene sheets to form coaxial cylinders. Single-walled carbon nanotubes (SWCNTs) and multi-walled carbon nanotubes (MWCNTs) correspond to one layer and more than one layers of rolled graphene, respectively [17]. The synthesis methods of CNTs mainly focus on arc discharge, laser ablation and chemical vapor deposition (CVD) [18]. Gr can be obtained through two approaches. One is the exfoliation of graphite, which contain mechanical exfoliation and chemical exfoliation, and the other is producing covalently linked two-dimensional networks by chemical reaction, such as epitaxial growth, pyrolysis and CVD [19]. 

Compared with graphite, whose thermal conductivity is always lower than or just around 100 W/m·K [9,11,12], the thermal conductivity of Gr can attain 5000 W/m·K that is even higher than about 3500 W/m·K of SWCNTs and about 3000 W/m·K of MWCNTs in the longitude direction [20,21]. This extraordinary superiority makes Gr and CNTs become promising candidates of conductive additives in the field of energy despite the high price. To promote the performance of thermosyphon devices or direct absorption solar collectors, Das et al. [22] and Zhang et al. [23] increased the thermal conductivity of nanofluids by incorporating Gr or CNTs. In the research presented by Amin et al. [24], Liu and Zhang [25], Karaipekli et al. [26] and Zhang et al. [27], the two carbon nanomaterials enhanced the thermal conductivity of phase change materials, which were mainly used to regulate temperature or reduce energy waste.

Some manifestations of Gr or CNTs in asphalt composites have been investigated over the last decade. The mixing of the carbon materials and asphalt always achieved by mechanical agitation, high-speed shearing and sonication depending on different demands. Faramarzi et al. [28] prepared MWCNTs modified asphalt binders by shear mixing process and wet process. The results showed that MWCNTs improved the rutting resistance as well as thermal cracking resistance of asphalt binders. Santagata et al. [29] stated that CNTs exhibited a positive influence on the fatigue properties of asphalt binders if a suitable dispersion technique was adopted. Moreno-Navarro et al. [30] proved that Gr modified asphalt binders could be heated more rapidly than matrix ones without weakening their stability. Gr and CNTs modification were found to be encouraging in enhancing the high-temperature properties, elastic recovery performance and pavement service life of asphalt binders from the investigation carried out by Yang et al. [31]. Li et al. [32] chiefly utilized the two carbon materials as promising microwave-absorbers to advance the self-healing properties of asphalt relying on microwave heating. Shirakawa et al. [33] demonstrated that CNTs increased stiffness along with the microwave absorption capability of asphalt emulsions. With respect to SBS copolymer modified asphalt binders, Shu et al. [34] reported their promotion in high-temperature anti-rutting properties and low-temperature anti-cracking properties with MWCNTs addition, and Goli et al. [35] probed their enhancement in storage stability. For application in asphalt concretes, Melo and Triches [36] carried out four-point fatigue tests and wheel tracking tests with the aim of evaluating the permanent deformation resistance of CNTs modified asphalt concretes, and the better mechanical performance indicated the effectiveness of CNTs.

Based on above review, although both Gr and CNTs have been used to increase the thermal properties of certain materials for down-to-earth energy applications, their actual thermal behaves in asphalt remains rarely explored. Moreover, although their favorable contribution to the promotion of some other properties of asphalt composites has been demonstrated, few reports have emphasized a direct comparison of their effects on asphalt composites and further analyzed the differences.

## 2. Scope of Work and Objectives

As essential thermal parameters of materials, thermal conductivity, thermal diffusivity and volumetric specific heat capacity can influence various thermal performances of functional asphalt pavements, including but not limited to solar harvesting efficiency, snow melting time, heat transfer speed and perpendicular temperature gradient [2,8,10]. Therefore, this study conducted quantitative analysis for the effects of Gr and CNTs on the three thermal parameters of asphalt binders. Gr and CNTs were used as thermal conductive additives, which also can be referred to as “modifiers” considering their feasibly low contents in asphalt binders. Meanwhile, the physical and rheological properties of the same modified asphalt binders were evaluated. Furthermore, combing with the experiments of modification mechanism, the influence factors resulting in different thermal conductive effect of Gr and CNTs were analyzed.

This study does not attempt to seek a situation in which the thermal, physical, and rheological performances are all optimized simultaneously, because the definition of the best optimization is maybe controversial according to actual circumstances and purposes. Instead, it aims to explore whether remarkable enhancement on thermal properties can be achieved by Gr or CNTs modified binders under the premise of meeting the basic usability of pavement construction, and hence judge their application prospects for solar harvesting and snow melting pavements.

## 3. Materials and Methods

### 3.1. Matrix Asphalt

Penetration grade matrix asphalt (90/100) was provided by Guochuang Co., Ltd., Wuhan, China. The measured values of the primary physical properties are listed in Table 1.

### 3.2. Graphene and Carbon Nanotubes

#### 3.2.1. Physical Parameters

**Figure 1:**
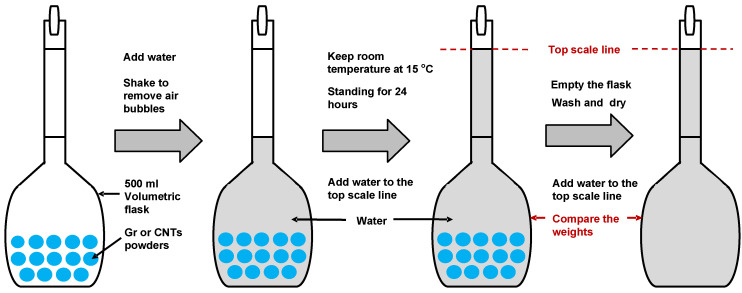
General view of measuring the apparent relative density of Gr and CNTs samples.

Graphene (Gr) produced by the mechanical exfoliation method and multiple-walled carbon nanotubes (CNTs) produced by the CVD method were supplied by Tuling Co., Ltd., Shenzhen, China. The physical parameters of them are shown in Table 2 and Table 3, respectively. It is worth noting that, to establish the relationship with previous studies applying carbon materials in thermal conductive asphalt concretes, this study followed the practice of employing the volume fraction of matrix asphalt to determine the modifier content, which was on the basis of the assumption that modifiers were used to substitute a portion volume of mineral powders in asphalt concrete. The relative density of the matrix asphalt compared with 15 °C water was already tested, and then the apparent relative density of Gr and CNTs samples in the same condition should be acquired to calculate the modifier weights corresponding to the targeted volume contents during preparing modified asphalt binders. This study consulted the “volumetric flask method” which is normally applied to measure the apparent relative density of fine aggregates in JTG E42-2005 [37]. The main procedure is generalized in Figure 1.

#### 3.2.2. SEM and HRTEM Observations

Microscopic morphologies of Gr and CNTs were detected by scanning electron microscopy (SEM) tests and high-resolution transmission electron microscopy (HRTEM) tests.

**Figure 2:**
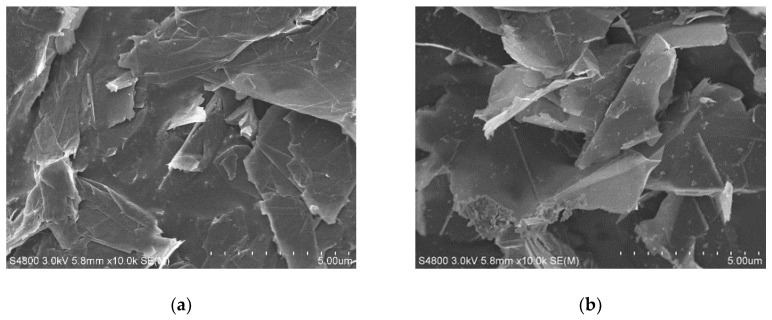

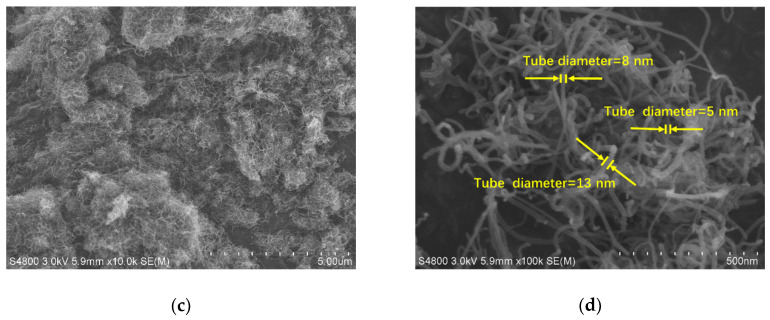
SEM images of Gr and CNTs: (**a**) Gr (5 μm scale bar, 10,000× magnification); (**b**) Gr (5 μm scale bar, 10,000× magnification); (**c**) CNTs (5 μm scale bar, 10,000× magnification); (**d**) CNTs (500 nm scale bar, 100,000× magnification).

Figure 2a,b show the SEM images of Gr, that exhibited a two-dimensional and sheet-like structure. The Gr sheets stacked together, although some of them bent slightly. When the scanning angle switched from the top view as shown in (a) to the side view as shown in (b), it was clear to see the interval space between each of the Gr sheets. This stacking status with interval spaces indicated that van der Waals forces between molecules of layer surfaces played the major role in Gr gathering. Figure 2c,d display SEM images of the CNTs, that exhibited a one-dimensional and tubular structure. The tubes were disorderly arranged and intertwined with each other. AN agglomeration phenomenon was obvious among the CNTs. Under the same magnifications, Figure 2a–c display that the radial size of the CNTs is at the nanometer scale and it looks much smaller than the micro-meter size of the plane of Gr sheet.

**Figure 3:**
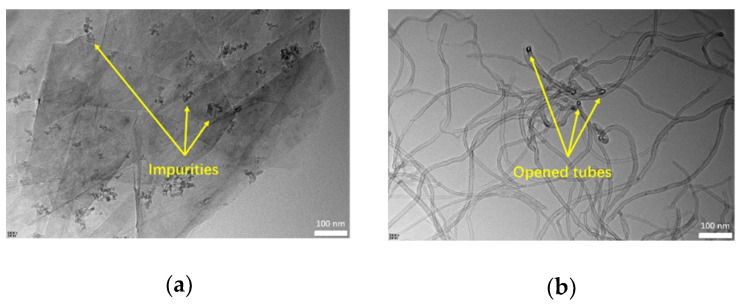
HRTEM images of Gr and CNTs: (**a**) Gr (100 nm scale bar, 50,000× magnification); (**b**) CNTs (100 nm scale bar, 50,000× magnification).

Figure 3 shows the HRTEM images of Gr and CNTs. The Gr presented a thin sheet-like structure, and there were a number of irregularly shaped impurities on it. For the CNTs, the opened tubes can be observed clearly. 

#### 3.2.3. Raman Scattering

**Figure 4:**
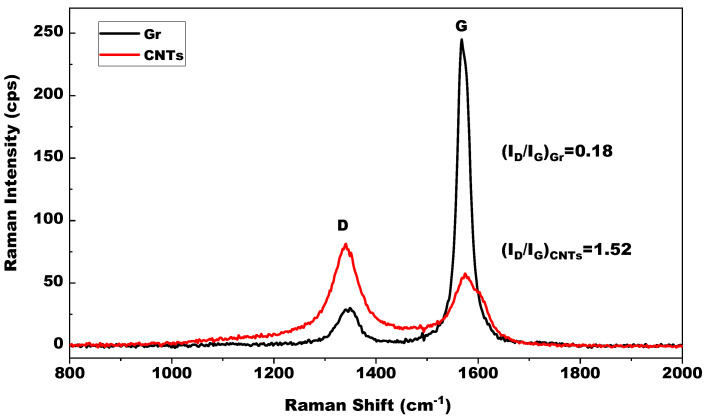
Raman spectra of Gr and CNTs.

Raman spectroscopy was used to investigate the composition, crystalline quality and defect level of Gr and CNTs samples. The characteristic peaks of their Raman spectra confirmed their identity of carbon, as illustrated in Figure 4. The strong peaks at 1567.82 cm^−1^ of Gr Raman spectrum and 1574.63 cm^−1^ of CNTs spectrum correspond to the typical “G” band of carbon material, which marks the in-plane vibration of sp^2^ hybrid structures in the layer, and the other strong peaks at 1349.17 cm^−1^ of Gr Raman spectrum and 1340.79 cm^−1^ of CNTs spectrum correspond to the disorder-induced “D” band of carbon material [38,39].

The intensity ratio of the “D” and “G” bands (I_D_/I_G_) calculated from the peak areas can judge the defects of Gr and CNTs [39]. For Gr sample, the I_D_/I_G_ ratio is equal to 0.18, which testifies the good quality of Gr with very few defects. For CNTs sample, the I_D_/I_G_ ratio is equal to 1.52, which indicated an inferior graphitization of it with more disordered carbon atoms than Gr.

### 3.3. Gr and CNTs Modified Asphalt Binders

#### 3.3.1. Preparation Samples

Gr modified asphalt binders (Gr-MA) and CNTs modified asphalt binders (CNTs-MA) were prepared via blending the modifiers and matrix asphalt at five percentages of modifiers (1%, 2%, 3%, 4% and 5%) by volume of matrix asphalt. The procedure consisted of three steps: First, 200 g of matrix asphalt was poured into an aluminum cup, then gradually heated to 145 °C and maintained at this temperature. Second, Gr or CNTs powder was put in and manually blended to asphalt. Third, the compound was stirred with a mechanical agitator at a speed of 500 rpm for 60 min to ensure a homogeneous mix. Looking forward to a well estimation on their application in the large-scale road construction, which should consider practicality and cost, this study did not adopt high-speed shearing or sonication in the mixing step. A contrast matrix asphalt binder was conducted with the same blend process in order to establish a reasonable comparison. 

#### 3.3.2. HRTEM Observations

**Figure 5:**
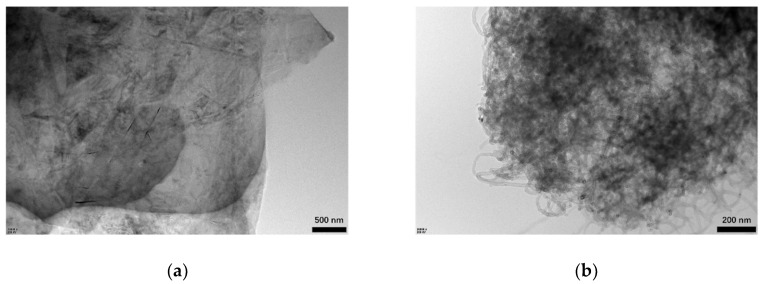
HRTEM images of asphalt binders modified by Gr and CNTs: (**a**) Gr-MA (500 nm scale bar, 10,000× magnification); (**b**) CNTs-MA (200 nm scale bar, 30,000× magnification).

Figure 5 displays the HRTEM images of 3% Gr-MA and 3% CNTs-MA. Compared with the images of raw Gr and CNTs in Figure 3, it can be observed that, overall, the color of the solid regions is blacker, which indicates the existence of asphalt. The blacker color presents the larger amount of asphalt covers Gr or CNTs. The CNTs agglomeration in asphalt can be observed in Figure 5b obviously. 

### 3.4. Thermal Properties Tests

**Figure 6:**
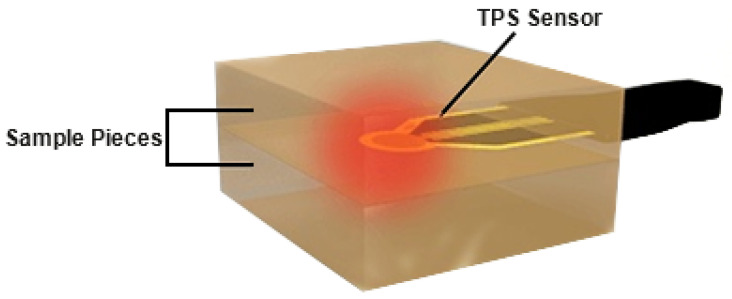
Working schematic diagram of the TPS sensor.

A thermal constants analyzer (Hot Disk TPS 2500S) was utilized to determine the thermal conductivity and thermal diffusivity of asphalt binders depending on the transient plant source (TPS) method. One binder was divided into two cuboid specimens in 50 mm length, 50 mm width and at least 20 mm height. The TPS sensor, which functioned as both the heating source and dynamic temperature probe [3], was sandwiched between the specimens as shown in Figure 6. They were settled and the thermal insulation was kept for at least 20 min to uniformize the temperature of the sensor and samples before each measurement. The room temperature was controlled at 20 °C throughout the whole process. 

To match the specimen size, the chosen TPS sensor type in this study was 4853, whose dimeter is 9.868 mm, depending on adjustments in advance. The test parameters such as electric power and measuring time also should be adjusted based on the thermal diffusivity of sample in order to satisfy the assumption that the sensor is situated in an infinite medium. For each of the two divided specimens, one measurement was recorded at one contact surface, and the other measurement was recorded at the opposite contact surface, respectively. The mean value of them was used as the result. It was infeasible to conduct repeated tests at one surface of the two divided specimens because duplication heating would soften the specimens seriously, especially for the same side, and made them fail to maintain the practicable height matching the adjusted test parameters. Besides, to prevent the sensor from breaking during rinsing off the adhesive asphalt, the contact surface of each specimen with the sensor was covered with a commercially available plastic wrap during the tests. It was reasonable because exploratory experiments found that there was no distinct discrepancy between the resultant data of the covered cuboid specimens and the uncovered ones.

On the grounds of definition, the volumetric specific heat capacity of asphalt binders could be calculated from Equation (1):

(1) $$C_{v}=\frac{\lambda }{\alpha }$$

where $C_{v}$ is the volumetric specific heat capacity; λ is the thermal conductivity; and α is the thermal diffusivity [40].

### 3.5. Physical Properties Tests

Penetration tests at 15, 25 and 30 °C, ring-and-ball softening point tests and ductility tests at 15 °C were carried out on asphalt binders in accordance with JTG E20-2011 [41].

Reflecting the temperature susceptibility of the asphalt binders, the penetration index (*PI*) was calculated through the penetration-temperature coefficient (*A_lgPen_*) obtained from the logarithmic relationship between the penetration values and temperatures, as Equations (2) and (3) noted.

(2) $$lgP=K+A_{lgPen}\times T$$

(3) $$PI=\frac{20-500A_{lgPen}}{1+50A_{lgPen}}$$

where *P* is the penetration; *T* is the test temperature; *K* and $A_{lgPen}$ are the constant term and the coefficient term of linear regression equation, respectively. 

*T*_800_, being named as the equivalent softening point, was computed as Equation (4). It is a high-temperature property index which is deduced to be equal to the temperature when penetration of asphalt binder reaches to 80 mm.

(4) $$T_{800}=\frac{lg800-K}{A_{lgPen}}$$

### 3.6. Rheological Properties Tests

#### 3.6.1. Brookfield Viscosity Tests

A Brookfield rotational viscometer (THERMOSEL DV-II+Pro) was utilized to measure the apparent viscosity of asphalt binders at 115, 135, 155 and 175 °C. Linear regression analysis on the interrelation between apparent viscosity and temperature acted up to the Saal equation as:

(5) $$lglg\left(\eta \times 10^{3}\right)=n-m\times lg\left(T+273.13\right)$$

where η is the apparent viscosity; *T* is the temperature; *n* and *m* are the constant term and coefficient term of equation, respectively. The slope of the regression line is defined as the viscosity temperature susceptibility (VTS) of the asphalt binders. 

#### 3.6.2. Dynamic Shear Rheometer (DSR) Tests

A dynamic shear rheometer (ANTON PAAR MCR101) was utilized to assess the rheological properties of asphalt binders according to AASHTO T315-09 [42].

High temperature sweep tests from 30 to 80 °C and low-to-intermediate temperature sweep tests from −10 to 30 °C were conducted in a controlled-strain testing mode at a frequency of 10 rad/s. Complex shear modulus (G*) and phase angle (δ) of asphalt binders were recorded.

A set of frequency sweep tests from 400 to 0.1 rad/s was proceeded at 20, 25, 30, 40, 50 and 60 °C. Fitted the complex modulus master curves of asphalt binders via introducing shift factors (*α_T_*) explained by Equation (6):

(6) $$E\left(T,t\right)=E(T_{0},\;t/\alpha_{T})$$

where *T* is the experiment temperature, *T*_0_ is the reference temperature, *t* is the objective parameter to be analyzed, *α_T_* is the shift factor. In this study, *t* is the complex shear modulus of asphalt binder, and *T*_0_ is 30 °C.

### 3.7. Modification Mechanism Tests

#### 3.7.1. Fourier Transform Infrared Spectroscopy (FTIR) Characterizations

A Fourier transform infrared spectrometer (Nicolet 6700) and a customized KBr crystal plate were adopted to explore the functional groups variation of asphalt binders after modification. Asphalt binder was dissolved in carbon disulfide to obtain a 5 wt. % solution. Two drops of solution were dripped onto the KBr crystal plate. The plate was exposed under a high-pressure mercury lamp for about 2 min until a thin film of asphalt remained. Then, the plate was inserted into the spectrometer and scanned at the wavelengths ranging from 4000 cm^−1^ to 500 cm^−1^, a resolution of 4 cm^−1^ and a scan number of 64. The original KBr crystal plate was scanned in advance at the same test parameters. 

#### 3.7.2. SARA Fraction Tests

A thin layer of chromatography with flame ionization detection (TLC-FID) (IATRON MK-6) was utilized to analyze the effect of modification on SARA fraction (saturates, aromatics, resins and asphaltenes) of the asphalt binders. A solution prepared by dissolving 80 mg asphalt binder in 4 mL dichloromethane. In total, 1 μL of solution was dotted by glass point capillary onto the original point of a chromatographic rod covered with silica gel. A set of ten rods for each binder was immersed in three developing tanks which respectively combine n-heptane, methylbenzene-n-heptane (volume ratio 4:1) and methylbenzene-absolute ethyl alcohol (volume ratio 9:11) in a sequence of ascending polarity [43,44]. The separating situation of the SARA components was judged through observation of the developing heights (100, 50 and 25 mm) of solution on the chromatographic rods. Consequently, quantitative analysis was processed via a flame ionization detector at a hydrogen flow of 160 mL/min, an air flow of at least 1.5 L/min and a scan speed of 30 s/column.

Presenting the sol-gel type of the asphalt binder, the collide instability index (*CII*), was defined by Equation (7) to evaluate the stability of the collide structure [45]:

(7) $$CII=\frac{A_{sp}+S}{R+A}$$

where *A_sp_*, *S*, *R* and *A* are the weight percentage of asphaltenes, saturates, resins and aromatics in asphalt binder.

## 4. Results and Discussion

### 4.1. Thermal Properties of Asphalt Binders

#### 4.1.1. Thermal Conductivity

**Figure 7:**
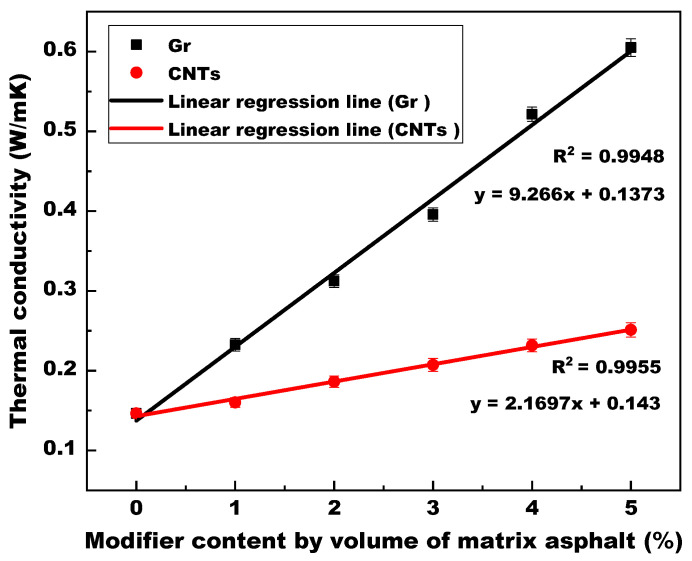
Thermal conductivity of asphalt binders modified by Gr and CNTs.

Figure 7 illustrates the influence of Gr and CNTs on the thermal conductivity of asphalt binders at 20 °C. The interrelations between thermal conductivity and modifier content were analyzed by linear regressions. For both Gr-MA and CNTs-MA, thermal conductivity increased with increasing modifier content in a favorable linear relationship, with a correlation coefficient (R^2^) of 0.9948 for Gr-MA and 0.9955 for CNTs-MA. In comparison, it was found that the effect of Gr was greatly superior to CNTs. When the modifier content increased from 0% to 5%, the thermal conductivity of Gr-MA was enhanced from 0.1465 to 0.6051 W/(mK) by 313.04%, while that of CNTs-MA increased to 0.2513 W/(mK) by 71.54%. The transportation of thermal properties in nanoscale carbon materials is primarily governed by phonons behavior [46,47]. Although both Gr and CNTs have high intrinsic thermal conductivity, the big gap between the thermal conductivities of asphalt binders modified by them mainly attribute to their specific surface area. Enlarging the interface between modifier and matrix asphalt, CNTs with larger specific surface area in this study enhanced the interface thermal resistance and strengthened the phonon scattering [48,49]. 

A higher thermal conductivity means that more heat can transfer across a unit distance through a unit sectional area inside the asphalt binder per unit time. Therefore, as solar heat collector, asphalt pavement adopting Gr-MA or CNTs-MA, especially Gr-MA, can harvest more solar energy from pavement surface and deliver more of it to heat the deep embedded pipes. 

#### 4.1.2. Thermal Diffusivity

**Figure 8:**
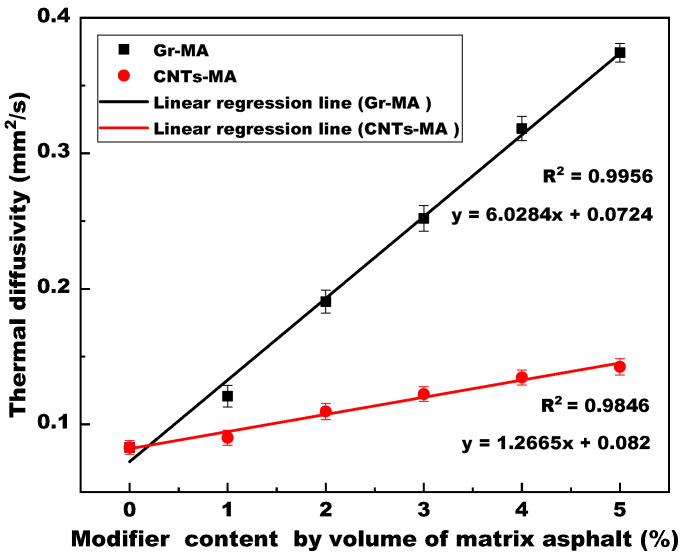
Thermal diffusivity of asphalt binders modified by Gr and CNTs.

Figure 8 shows the linear regression analysis for the thermal diffusivity of modified asphalt binders with varying modifier content at 20 °C. Being analogous with the variation tendency of thermal conductivity, the thermal diffusivity of both Gr-MA and CNTs-MA linearly increased with the increase of modifier content, and the correlation coefficients (R^2^) were 0.9956 and 0.9846, respectively. Incorporating 5% Gr in asphalt binder contributed to a significant thermal diffusivity gain by 350.84%, whereas the thermal diffusivity of 5% CNTs-MA reached 0.1424 mm^2^/s with a 71.57% increment. A higher thermal diffusivity, which is a more visualized thermal parameter compared with thermal conductivity, signifies a faster temperature transfer in asphalt binders. 

#### 4.1.3. Volumetric Specific Heat Capacity

Owing to the satisfactory consequence of linear regression on thermal conductivity and thermal diffusivity, this study calculated the volumetric specific heat capacity ($C_{v}$) of Gr-MA and CNTs-MA according to Equation (1) through dividing the linear regression equation of thermal conductivity by that of thermal diffusivity as Equations (8) and (9), instead of directly recording the values measured by HOT DISK thermal constants analyzer.

(8) $${\left(C_{v}\right)}_{Gr-MA}=\frac{9.266x+0.1373}{6.0284x+0.0724}$$

(9) $${\left(C_{v}\right)}_{CNTs-MA}=\frac{2.1697x+0.143}{1.2665x+0.082}$$

where ${\left(C_{v}\right)}_{Gr-MA}$ and ${\left(C_{v}\right)}_{CNTs-MA}$ are the volumetric heat capacity of Gr-MA and CNTs-MA.

**Figure 9:**
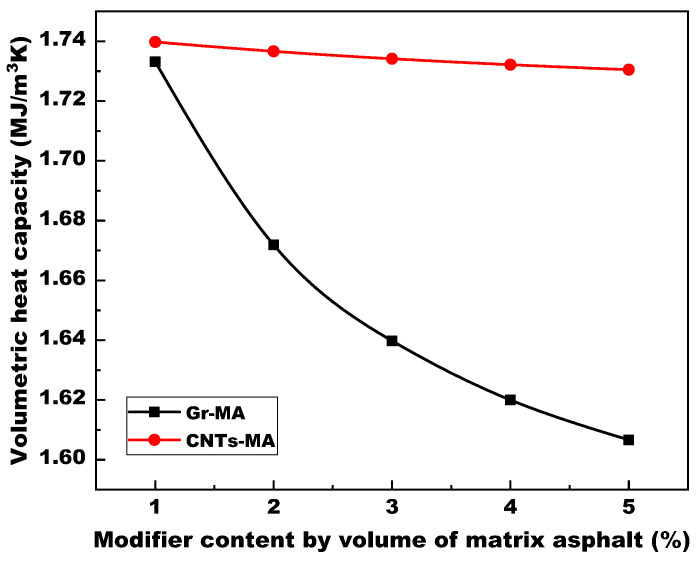
Volumetric specific heat capacity of asphalt binders modified by Gr and CNTs.

As shown in Figure 9, ${\left(C_{v}\right)}_{Gr-MA}$ and ${\left(C_{v}\right)}_{CNTs-MA}$ experienced varying degrees of decline as the modifier content increased. 5% modification decreased ${\left(C_{v}\right)}_{Gr-MA}$ to 1.6067 MJ/m^3^ K and decreased ${\left(C_{v}\right)}_{CNTs-MA}$ to 1.7305 MJ/m^3^ K. According to the definition of $C_{v}$ which indicates the requisite heat absorbs by a unit volume of objective material for supporting 1 K rise of temperature, Gr-MA will consume less heat than CNTs-MA to ascend to an identical temperature. In other words, for Gr-MA, because 1 K warming up absorbs less heat, more remnant heat continues to be transported inward the binder to even-up the temperature at every point of binder eventually faster, compared with CNTs-MA. Interrelated with each other, volumetric specific heat capacity and thermal diffusivity determine the temperature propagation velocity inside asphalt binder.

To sum up, the quantitative analysis on the three important thermal parameters revealed that the 5% Gr and 5% CNTs modifications resulted in more than 3 times and about 0.72 times increase respectively in both thermal conductivity (λ) and thermal diffusivity (α) of asphalt binders at 20 °C. Furthermore, combined with the decreasing trend of volumetric specific heat capacity$\;(C_{v})$, for solar-harvesting pavements, the two modifiers can not only assist the asphalt concrete in gaining more solar energy from the pavement surface, but also accelerate the pace of absorbed heat as well as temperature transferring from pavement surface to the embedded pipes. Therefore, the solar harvesting efficiency can be improved and the UHI effect will be mitigated consequently. In turn, emphasizing on the process of heat transport from embedded pipes to pavement surface, the snow melting pavements also need these two thermal conductive modifiers to reduce the snow melting time and thus achieve efficiency progress. By comparison, Gr exhibited better modification effects on thermal properties of asphalt binder. 

### 4.2. Physical Properties of Asphalt Binders

#### 4.2.1. Penetration and Temperature Susceptibility

**Figure 10:**
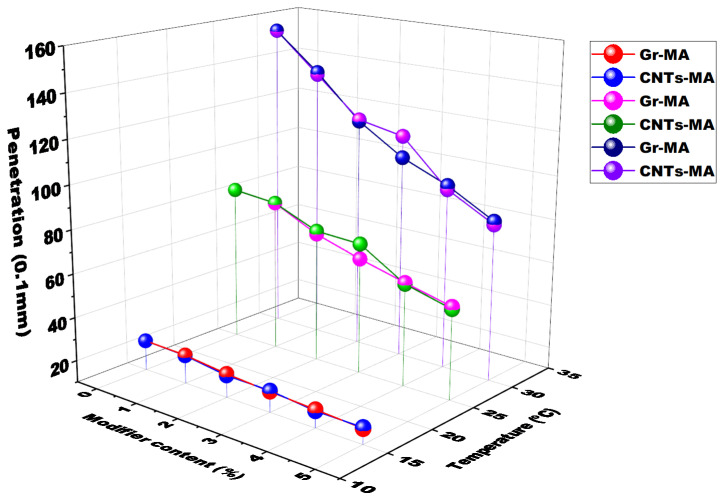
Penetration of asphalt binders modified by Gr and CNTs at different temperatures.

Figure 10 presents the penetration change of Gr-MA and CNTs-MA at three moderate temperature including 15 °C, 25 °C and 30 °C, along with increasing the modifier content from 0 to 5%. The penetration test is always used as a measurement of consistency. A higher penetration degree indicates softer consistency of asphalt binder [50]. As illustrated, the addition of Gr or CNTs resulted in a harder consistency of binder. This attributed to the dispersion of modifiers with greater density in asphalt binder [51].

Unlike Gr-MA whose penetration degree decreased steadily with the increase of Gr content, for CNTs-MA, the penetration degrees of 2% CNTs-MA and 3% CNTs-MA were close, and the value suddenly decreased remarkably after the modifier content surpassed 3%. This trend owed to two reasons. For one thing, agglomeration of CNTs in 3% CNTs-MA reduced the modification effect. For another, because of the large specific surface area of CNTs, the great surface energy resulted in strong interfacial interaction between CNTs and asphalt, which made the consistency of binder become harder [52,53]. When CNTs content increased to 4% and 5%, compared with agglomeration, the interfacial interaction played a more influential role in the modification effect on penetration. Then, the penetration degree of CNTs dropped sharply.

The penetration index (PI) is a commonly adopted index to represent the temperature susceptibility (TS) of asphalt binders. Greater PI signifies that the binder is less sensitive to temperature and performs better high-temperature properties. The binder whose PI < −2 approaches to the Newtonian fluids and manifests the sol-type characteristic with quite high TS. It cannot be used for pavement. The binder whose PI > 2 manifests the gel-type characteristic. It deviates from Newtonian fluids in a large extent and owns good high-temperature properties but terrible cracking resistance at low temperatures. 

**Figure 11:**
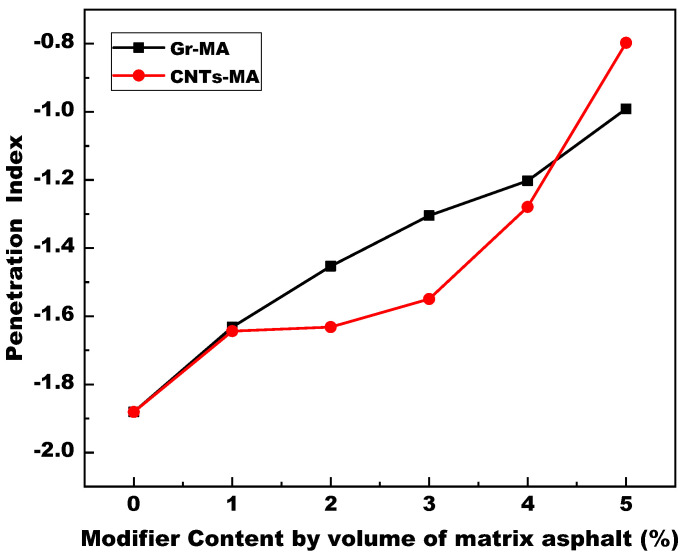
Penetration index (PI) of asphalt binders modified by Gr and CNTs.

Figure 11 illustrates the PI results of Gr-MA and CNTs-MA. The PI values all located between −2 and 2 where corresponded to the sol-gel type of asphalt binders. The TS was moderate for pavement application [53]. With the addition of Gr or CNTs, asphalt binders became less sensible to elevating temperature and obtained better high-temperature properties. Moreover, except 5% dosage, PI values of Gr-MA were all greater than that of CNTs-MA. For CNTs-MA, the marginally PI change between 1 to 3% dosage also pointed out the existence of agglomeration phenomena among CNTs at moderate temperature range. 

#### 4.2.2. Softening Point and Equivalent Softening Point

**Figure 12:**
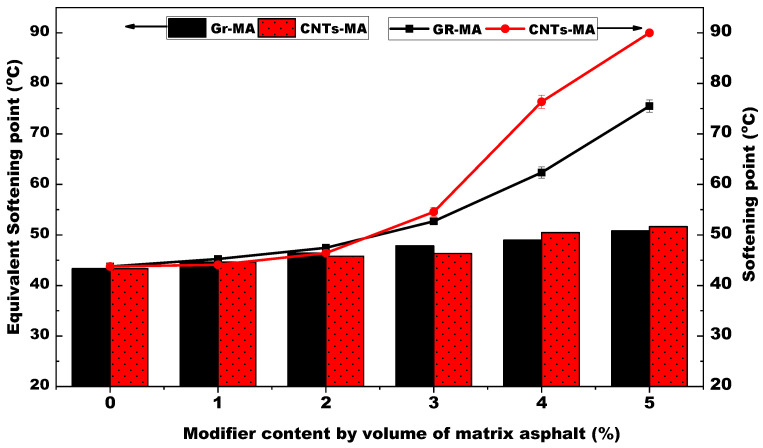
Softening point and equivalent soft point of asphalt binders modified by GR and CNTs.

The softening point (T_R&B_) and equivalent soft point (T_800_) are both indexes to characterize the high-temperature stability of asphalt binders. Both Gr and CNTs contributed to enhancing the high-temperature stability of asphalt binders, as shown in Figure 12. Since the layer structure of Gr or the intertwined tubular structure of CNTs obstructed the movement of asphalt molecular chains at high temperature, the binders’ stability against flowing was promoted.

Additionally, because the thermal motion of asphalt molecular chains is more active with an increase of temperature, the impaction of obstructed molecular movement after incorporating modifiers on binders’ stability would manifest more obviously at higher temperatures. As a result, for the majority of Gr-MA and CNTs-MA samples, T_R&B_ which was tested through elevating temperature to more than 40 °C was higher than T_800_ which was calculated from the penetration of samples at moderate temperature no more than 30 °C.

For 1% CNTs-MA, its T_R&B_ was lower than T_800_. This phenomenon could be explained by the experiment circumstance. While a penetration test was proceeded at a constant temperature involving no thermal properties of CNTs, the ring-ball softening point test was conducted in distilled water whose temperature raised persistently in a constant velocity. CNTs with high thermal conductivity accelerated the warming up process insider binder. Then, neglecting the obstructed molecular movement on account of low modifier content, asphalt binder could be soft enough to allow the test ball falling at a temperature before the penetration degree was supposed to be 800.

#### 4.2.3. Ductility of Modified Asphalt Binders

**Figure 13:**
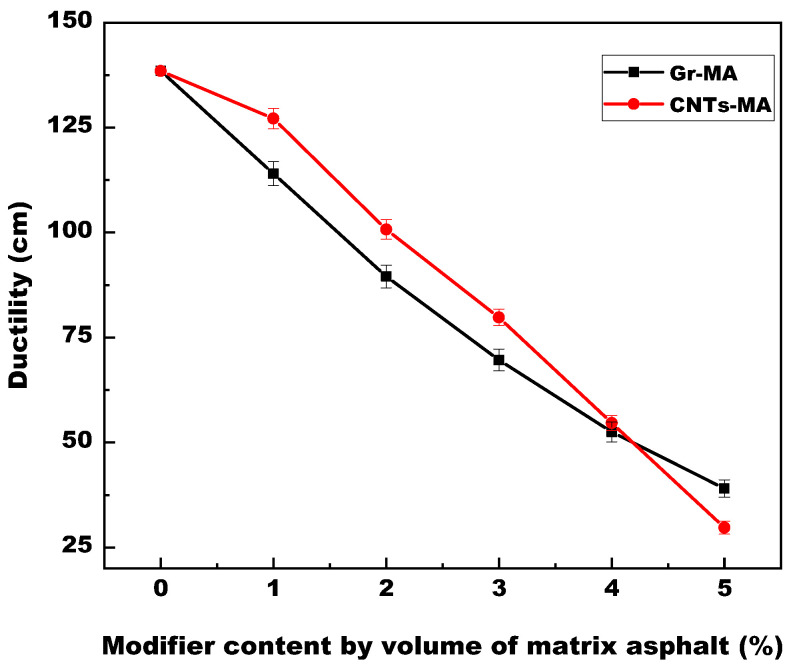
Ductility (15 °C) of asphalt binders modified by Gr and CNTs.

Asphalt binder with greater ductility value shows better adherence, which is beneficial to coating aggregates, and is more resistant to cracking at low temperatures consequently [54]. As illustrated in Figure 13, incorporating Gr or CNTs dramatically reduced the 15 °C ductility of binder by 71.8 or 78.7%, respectively. This was mainly because hardening consistency accelerated the fracture process of asphalt binders when it countered tensile stress [52]. The fact that the relationship between ductility values of Gr-MA and CNTs-MA at the same dosage was completely on the contrary of their 15 °C penetration degree also certified the influence of consistency on ductility. Besides, except for at 5% dosage, CNTs-MA were more ductile than Gr-MA, probably attributing to the “pull-out” behavior of CNTs which strengthened the adhesion of their interphase to a certain extent [55]. Generally, both Gr and CNTs reduced the low-temperature anti-cracking property of asphalt binders.

### 4.3. Rheological Properties of Asphalt Binders

#### 4.3.1. Brookfield Viscosity and Viscosity Temperature Susceptibility

**Figure 14:**
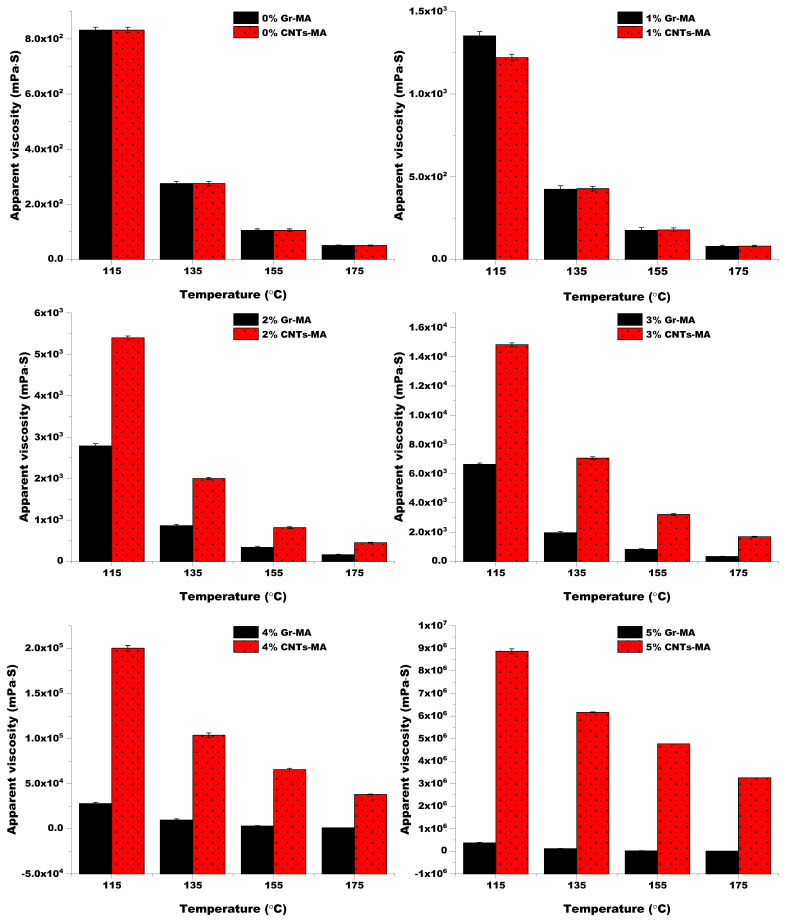
Apparent viscosity of asphalt binders modified by Gr and CNTs at different temperatures.

Figure 14 synthesizes the influence of Gr and CNTs on the apparent viscosity of asphalt binders, which is the ratio of shear stress to shear rate for a Newtonian liquid or a non-Newtonian liquid [56]. Apparent viscosity is also deemed as an indicator of the high-temperature properties of asphalt binders. As displayed, the increase in Gr and CNTs content increased the apparent viscosity of asphalt binders, that was because the movement restriction of asphalt molecular chains and strong interfacial interaction between modifiers and asphalt [57,58] enhanced binder’s resistance to flow during the temperature elevation. Besides, CNTs-MA accomplished 1–260 times higher apparent viscosity than Gr-MA at the experimental temperatures when the modifier content was increased from 2% to 5%. This was ascribed to the larger specific surface area of CNTs, which led to a stronger interfacial interaction with asphalt. 

The apparent viscosity at 135 °C is a significant index which should not be above 3 Pa·s on the basis of the SHRP (Strategic Highway Research Program) criteria for guaranteeing the work ability of asphalt binder during pavement construction [59]. With the addition of modifier, the dosage which originally generated an apparent viscosity exceeding 3 Pa·s was between 3% and 4% for Gr-MA, and it was between 2% and 3% for CNTs-MA. Thereby, in this study, 4–5% Gr-MA and 3–5% CNTs-MA cannot be applied for solar harvesting and snow melting pavements.

For 3% Gr-MA and 2% CNTs-MA, first, their apparent viscosities were quite close. Second, for thermal parameters, the thermal conductivity and thermal diffusivity of 3% Gr-MA at 20 °C were 0.4153 W/Mk and 0.2533 mm^2^/s derived from the fitting out equations of linear regression, respectively, which were both higher than that of 2% CNTs-MA, and the volumetric specific heat capacity of 3% Gr-MA at 20 °C was 1.6398 MJ/m^3^ K calculated from Equation (8), which was lower than that of 2% CNTs-MA. These were all beneficial to the work efficiency of solar harvesting and snow melting pavements. Third, in terms of physical properties, compared with 2% CNTs-MA, 3% Gr-MA had lower penetration degree, higher penetration index and higher softening point, demonstrating superior high-temperature properties. However, 3% Gr-MA was less ductile than 2% CNTs-MA, which signified inferior low-temperature properties. Further comparisons will be proceeded in the following analysis concentrating on other experiments.

**Figure 15:**
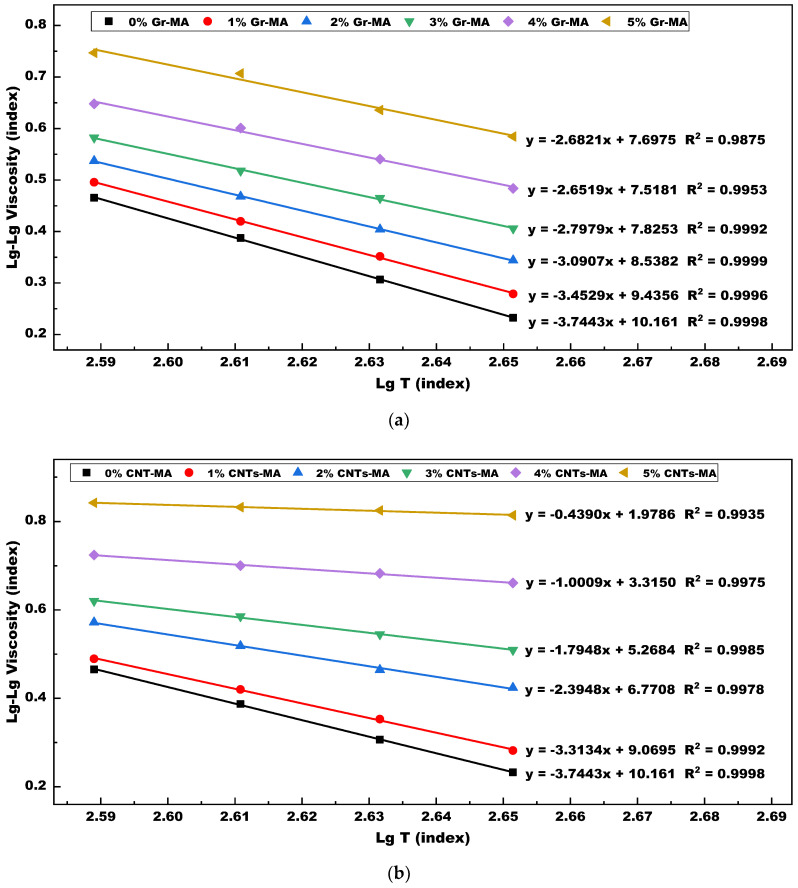
Lg-Lg Viscosity versus Lg Temperature with different content of Gr and CNTs modified asphalt binders: (**a**) Gr as modifier; (**b**) CNTs as modifier.

**Figure 16:**
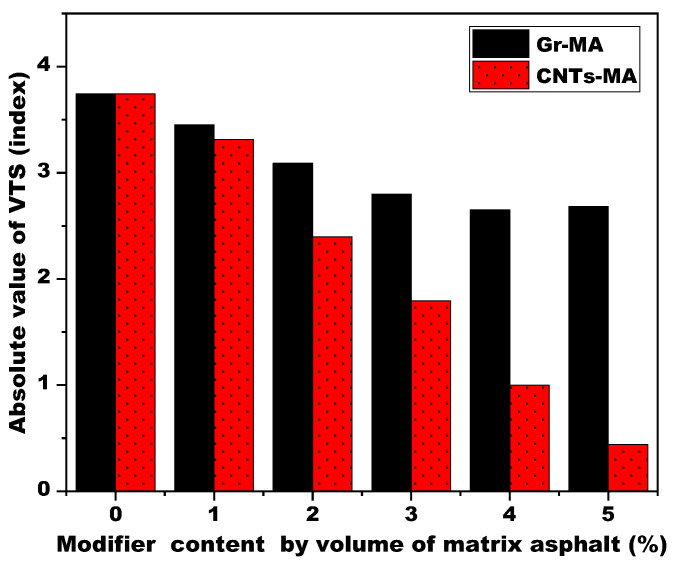
Viscosity temperature susceptibility (VTS) of asphalt binders modified by Gr and CNTs.

A general overview of viscosity-temperature equations and viscosity temperature susceptibility (VTS) of Gr-MA and CNTs-MA is provided in Figure 15. VTS itself is a negative. The lower the absolute value of VTS means that asphalt binder is less sensitive to temperature, or rather owns lower temperature susceptibility (TS). Figure 16 intuitively reflects the comparison on the TS changes of Gr-MA and CNTs-MA. As shown, incorporating CNTs caused a remarkable descent of the TS of asphalt binder by 88.28% and the descend rate did not fluctuate too much during the modifier content increase. However, for Gr-MA, although the overall trend of TS change was reduction, the change rate was smaller than the CNTs one. Especially when Gr content increased above 3%, the variation turned to be marginal. Then, keeping the continued incorporation of Gr would not be quite significant to the TS reduction and high-temperature properties improvement of asphalt binders. This reinforces the notion that 3% is a good dosage for Gr-MA. Besides, compared with 3% Gr-MA, 2% CNTs-MA manifested lower TS. 

Compared with PI, which is another TS indicator discussed above, the TS altering trends were not always the same. First, while for PI, TS of Gr-MA was lower than CNTs-MA at the modifier contents from 1 to 4%, TS derived from VTS manifested that Gr-MA was always more sensitive to temperature than CNTs-MA. Second, for Gr-MA, PI demonstrated a prominent TS decrease in a sustainable pace, but VTS showed a fast TS reduction at lower dosages initially and slowing down obviously at higher dosages. The discrepancy could be explained by the difference in temperature range. It was deducible that, from the perspective of reducing TS, CNTs performed better than Gr in high temperature range, and Gr was more effective in moderate temperature range. 

#### 4.3.2. Viscoelastic Properties in the High Temperature Range

Viscoelastic behavior of asphalt directly influences the pavement service property of asphalt concrete. During summer, asphalt performs more viscous feature which is the major cause of rutting at high temperatures. During winter, it performs more elastic features but may lead to cracking at low temperatures. 

**Figure 17:**
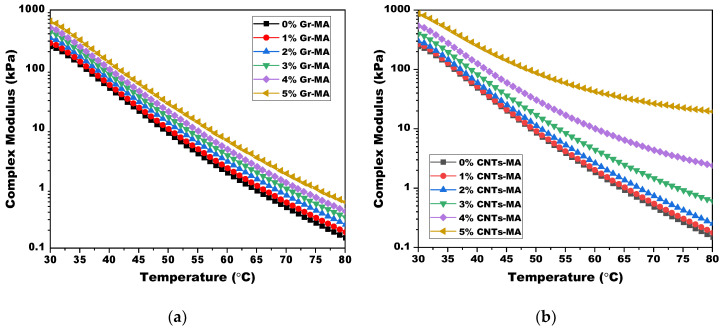
Complex modulus (G*) of asphalt binders modified by Gr and CNTs at high temperature range: (**a**) Gr as modifier; (**b**) CNTs as modifier.

The complex shear modulus (G*) represents the binder’s resistance to shear deformation under oscillatory load. It reflects the stiffness of asphalt binder [42]. Figure 17 illustrates the temperature dependence of G* of Gr-MA and CNTs-MA within the high temperature range from 30 to 80 °C. Originally, experimental results demonstrated that both two modifiers did not change the variation trend of G* from persistently deceasing with elevating temperature like the matrix binder behaved. Meanwhile, at an identical temperature, both Gr-MA and CNTs-MA turned to be stiffer, gaining better high-temperature properties with the increase of modifier content.

When the modifier content reached 3%, CNTs-MA became stiffer than Gr-MA. Especially when temperature raised more closely to 80 °C, the gap enlarged even bigger. This was consistent with the consequence obtained from the softening point test which was another characterization of high-temperature properties operated in the similar temperature range. Data comparison also drove to a find that, except 1% dosage, G* of CNTs-MA were all greater than that of Gr-MA at 80 °C, in agreement with the predicted behavior from the Brookfield viscosity tests at 115 °C. Combining the consistency of these two, it was concluded that CNTs-MA performed stronger resistance to shear deformation than Gr-MA either at high temperature or with high incorporating content.

**Figure 18:**
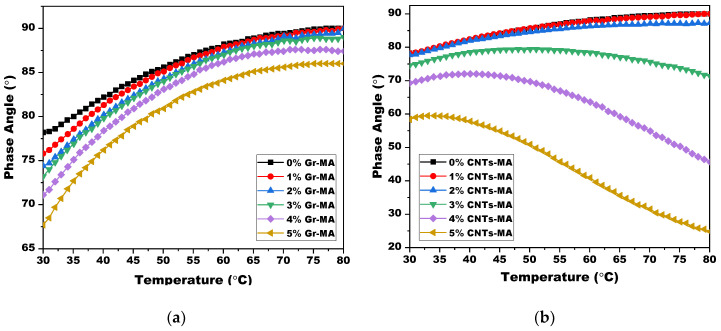
Phase angle (δ) of asphalt binders modified by Gr and CNTs in the high temperature range: (**a**) Gr as modifier; (**b**) CNTs as modifier.

Defined as the angle in radians between imposed stress and resulting strain, phase angle (δ) reflects the viscoelastic state of asphalt binders [42]. The smaller the δ is, the more elastic features are performed by the binder. The larger the δ is, the more viscous features are embodied, on the contrary. In Figure 18a, it was shown that δ of matrix binder and Gr-MA increased as temperature elevated. And the augment of Gr content led to a decrease of δ at an identical temperature, which indicated the elastic proportion enhancement of asphalt binder. In Figure 18b, although the variation tendencies of 1% CNTs-MA and 2% CNTs-MA were similar with that of matrix binder as well as Gr-MA with increasing temperature, once the dosage increased to 3% and more, δ of CNTs-MA increased momently and converted to decrease drastically. This phenomenon is analogous to the δ change of SBS modified asphalt binders. Therefore, it was reasonable to presume that an ample amount of CNTs built a network of nanotubes, which contribute to significant elasticity promotion at a comparatively high temperature [60]. 

**Figure 19:**
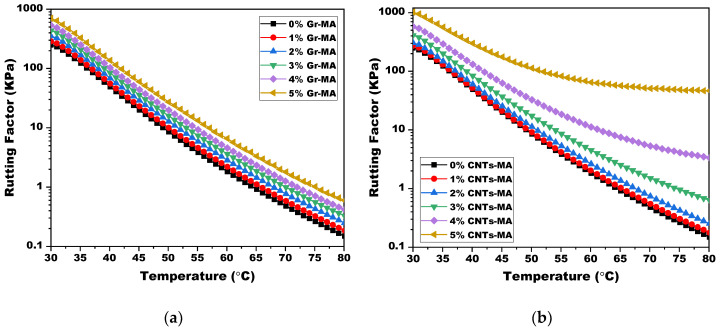
Rutting factor (|G*|/sinδ) of asphalt binders modified by Gr and CNTs at high temperature range: (**a**) Gr as modifier; (**b**) CNTs as modifier.

Rutting factor (|G*|/sinδ) is a performance-related parameter of asphalt binder, which indicates the capability to resist permanent deformation at high temperatures. The higher the |G*|/sinδ is, the smaller amount of energy dissipates during each load cycle, and the more high-temperature stiffness is gained [42]. The graphs in Figure 19 display the influence of Gr and CNTs on rutting factor of asphalt binders. For both of them, with the increase of dosage, binder’s potential on withstanding permanent deformation advanced. Thus, it was more difficult for rutting to appear on pavement under traffic load. In addition, the huge raise on |G*|/sinδ of 3–5% CNTs-MA at 80 °C also supported the judgement that a network of nanotubes was formed. 

In SHRP criteria, characterizing the high-temperature performance of asphalt binders directly, the performance grade (PG) relates to the temperature in which the |G*|/sinδ value of asphalt binder is greater than or equal to 1 KPa at an experimental frequency of 10 rad/s [59]. As listed in Table 4, 4 and 5% Gr addition elevated the PG from 64 to 70, whereas CNTs-MA experienced a more prominent PG enhancement up to 82 which was logically estimated with the variation tendency of rutting factor, in the situation where the upper limit temperature of the DSR tests in this study was 80 °C.

Finally, for 3% Gr-MA and 2% CNTs-MA, according to the detailed data, G* and |G*|/sinδ of 3% Gr-MA were always greater than those of 2% Gr-MA. When temperature was between 30 °C and 54.1 °C, δ of 3% Gr-MA was larger than that of CNTs-MA. When temperature was between 54.1 °C and 80 °C, CNTs-MA owns the larger δ. The high-temperature PG of them were both 64. Comprehensive analysis drew a conclusion that 3% Gr-MA owned better high-temperature rutting resistance at least between 30 °C and 80 °C.

#### 4.3.3. Viscoelastic Properties of Modified Asphalt Binders in the Low-to-Intermediate Temperature Range

**Figure 20:**
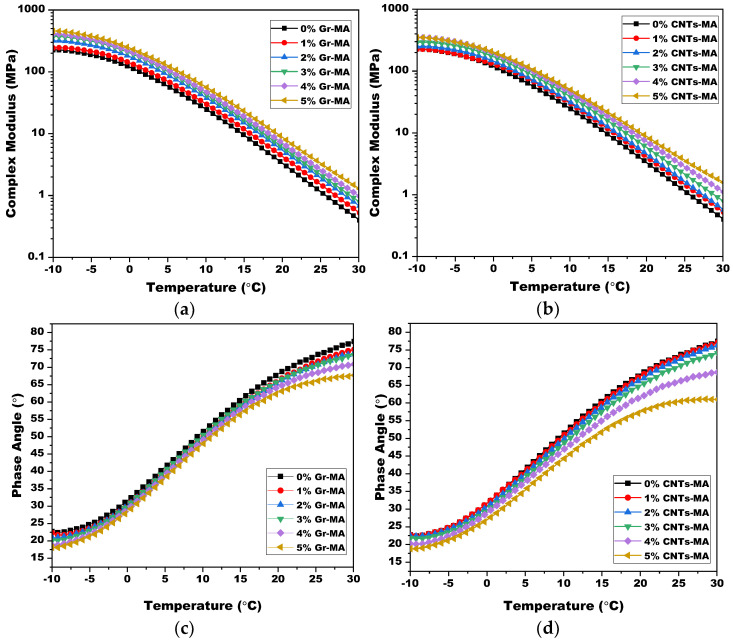
Complex shear modulus (G*) and phase angle (δ) of asphalt binders modified by Gr and CNTs in the low to intermediate temperature range: (**a**) G* of Gr-MA (**b**) G* of CNTs-MA; (**c**) δ of Gr-MA; (**d**) δ of CNTs-MA.

Figure 20 provides an overview of the temperature dependence of Gr-MA and CNTs-MA within the temperature range from −10 °C to 30 °C. As the graphic represents, with their effects resembling in the high temperature range, both Gr and CNTs resulted in the G* increase and the δ decrease of asphalt binders. However, as the temperature increased, the δ of both Gr-MA and CNTs-MA increased persistently even after the CNTs content surpassed 2%, which indicated that the CNTs network had not be formed in this temperature range.

According to detailed analysis on Figure 20, at 30 °C, G* of CNTs-MA was greater than that of Gr-MA at 1, 4 and 5% dosage, whereas 3% and 4% dosage offered the reverse. The relationships are accordant with their consistency correlation obtained from 30 °C penetration tests. Therefore, it is rational to deduce that the change of stiffness (a rheological property) and consistency (a physical property) are synchronous during modification. At −10 °C, except 1% dosage, δ of Gr-MA were all smaller than that of CNTs-MA. The more elastic binder at the edge of low temperature performed the poorer anti-cracking potential. In general, Gr contributed to more adverse reduction on the low-temperature performance of asphalt binders at a temperature around −10 °C. The comparison between effects of 3% Gr and 2% CNTs arrived at a conclusion that 3% Gr-MA had weaker cracking resistance than 2% CNTs-MA.

#### 4.3.4. Complex Shear Modulus Master Curves

**Figure 21:**
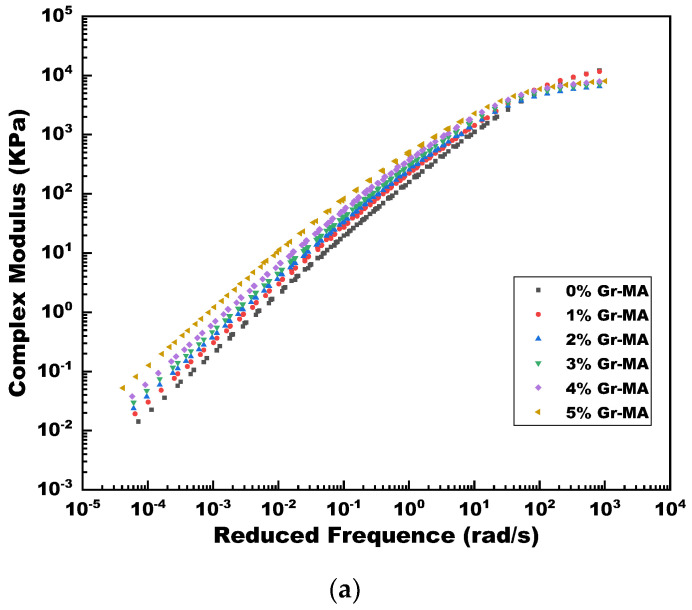

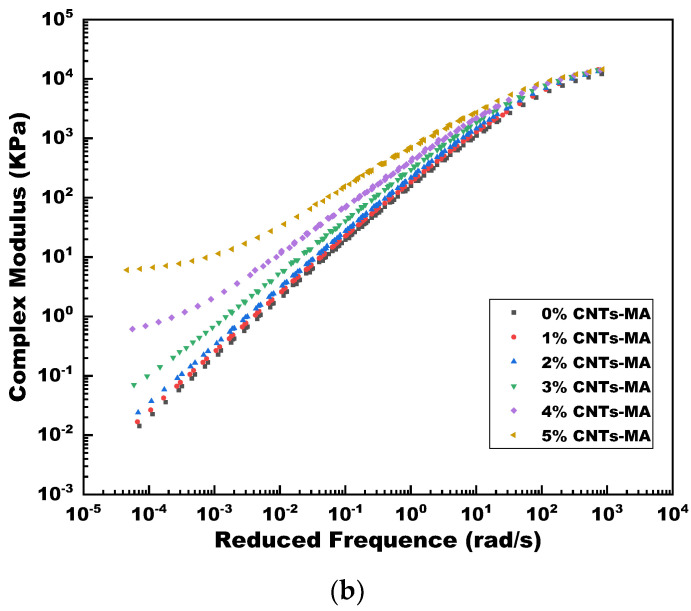
Complex modulus master curves of asphalt binders modified by Gr and CNTs. (**a**) Gr as modifier; (**b**) CNTs as modifier.

In terms of the time-temperature superposition principle, mechanical response of viscoelastic materials can be observed or estimated on condition of at lower temperature sustaining longer time or at higher temperature sustaining shorter time [61]. Figure 21 presents the complex shear modulus master curves of Gr-MA and CNTs-MA, which expand the frequency scope from 0.1–400 to 10^−5^–10^4^ rad/s according to Equation (6).

As illustrated, at extremely high temperature, both Gr and CNTs performed good continuity of enhancing the high-temperature performance of asphalt binders. CNTs also manifested better modification efficiency at higher temperatures and higher dosages. 

At extremely low temperature, while the stiffness of CNTs-MA increased with the increase of modifier content, that of Gr-MA presented in a different way. Originally, 1% and 2% Gr addition decreased the G* of asphalt binder from 12,100 kPa to 11,600 kPa and 6490 kPa, respectively. Subsequently, although G* converted to increase when Gr content reached 3%, it just increased to 8030 kPa of 5% Gr-MA at the final which was still smaller than the original 12,100 kPa. This phenomenon was probably due to the unique two-dimensional structure of Gr with strong hexagon lattice, resulting in quite weak binding force between the layers of stack, quite strong bearing capability perpendicular to the layers, and extraordinary smooth surfaces with low surface energy [62]. This crystal structure made Gr have excellent tribological potential [63], which reduced the cohesion among components of asphalt binder and accordingly gave rise to the stiffness reduction. Because the stiffness of the asphalt itself was great at extremely low temperature, it was detectable for G* reduction after dispersing a small amount of lubricative Gr into asphalt. Accompanied with the increasing Gr volume in the asphalt, the high Young’s modulus and great stiffness of Gr led to a modest recovery of binder’ G*.

**Figure 22:**
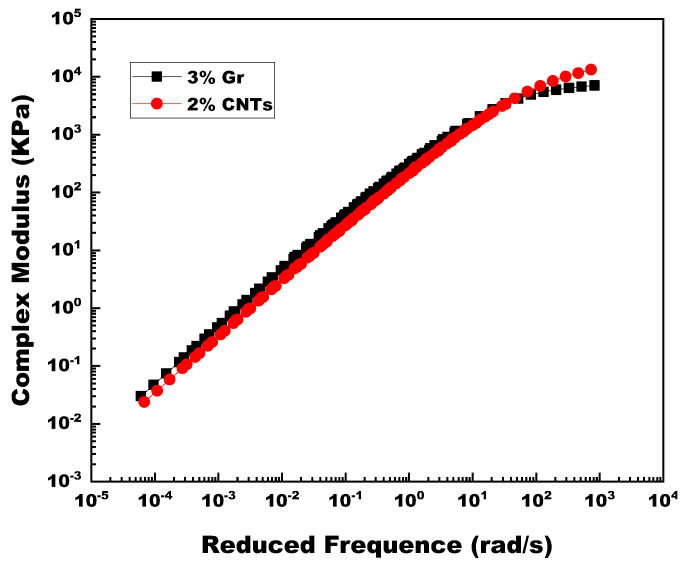
Complex modulus master curves of 3% Gr and 2% CNTs modified asphalt binders.

Moreover, it is crucial to compare the contribution of 3% Gr and 2% CNTs on asphalt binders in the broadest temperature range. The results displayed in Figure 22 show that 3% Gr-MA was stiffer at the extremely high temperature, and was softer at the extremely low temperature, which were both superior properties for the sake of impressive pavement performance no matter in high-temperature or low-temperature surroundings. 

### 4.4. Modification Mechanism of Gr and CNTs Modified Asphalt Binders

#### 4.4.1. Chemical Structure

**Figure 23:**
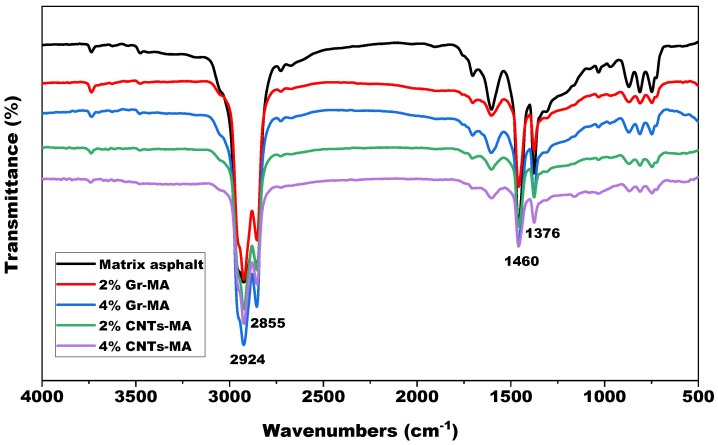
FTIR spectra of matrix asphalt binder and modified asphalt binders.

FTIR tests were conducted to explore the functional groups variation of asphalt binders after modification. The characterization can judge whether chemical reactions have occurred. Figure 23 shows the FTIR spectra of matrix asphalt, Gr-MA and CNTs-MA. As shown, the absorption peaks at 2924 cm^−1^ and 2855 cm^−1^ marks the CH_2_ stretching vibration, attributing to the existence of long chain alkyl. The absorption peaks at 1460 cm^−1^ and 1376 cm^−1^ marks the CH_3_ bending vibration because of the existence of CH_3_ functional group. The four intensive absorption peaks all correspond to the typical asphalt constitutions including alkanes and cycloparaffin. It is more essential that the positions of all distinct absorption peaks are coincident, and no other new peak appears in the spectrum of Gr-MA and CNTs-MA samples. Consequently, both two modifiers were physically blended with matrix asphalt and no detectable chemical reaction occurred during modification. That also reinforced the notion that the interface thermal resistance between the modifier and asphalt was probably a crucial factor which caused the wide gap between the thermal parameters of Gr-MA and CNTs-MA.

#### 4.4.2. SARA Fraction

Asphalt owns a colloidal attribute and is considered as a peptization of asphaltenes micelles by resins in an oily medium in which aromatics and saturates act as continuous phase in a continuous matrix. Saturates are mainly comprised by aliphatic chain compounds. Aromatics, resins and asphaltenes have progressive polarity and molecular mass, containing aromatic compounds with various heteroatoms [43,45]. SARA fraction impacts the stability of asphalt colloidal system and ulteriorly impact the performance of asphalt binders ulteriorly, according to the modern colloid theory.

Table 5 and Table 6 list the SARA fraction and collide instability index (*CII*) of Gr-MA and CNTs-MA. The greater the *CII* ratio, the more the asphalt is of sol type, and the higher the stability of asphaltenes is in the colloidal system [45,64]. As listed, *CII* was increased by 9.90% and 32.85% with the use of 5% Gr and 5% CNTs, respectively. As an inorganic substance, Gr or CNTs could not dissolve in the organic developers, and hence stayed at the original point of the chromatography rods instead of being developed to target heights. In the subsequent flame ionization process, they were detected as “asphaltene”. Gr and CNTs could not well peptized by the resins in the oily medium. Additionally, the strong interfacial interaction between asphalt and modifiers ascribed to the large specific surface area of modifiers disequilibrated the asphalt colloidal system which was organized and stabilized via continuous polarity of SARA components, forming non-covalent functional groups by absorbing molecules with aromatic structures onto the surface of modifier through π–π conjugation. Hence, no matter for Gr-MA and CNTs-MA, a portion of resin stayed with modifier at the original points of chromatography rods during the experiments. That is another reason why the weight fraction of resin showed a downward trend with augment of asphaltenes. In summary, Gr and CNTs modification weakened the stability of asphalt binders, promoted their gelation, and improved their elasticity. CNTs-MA suffered from more seriously gelation than Gr-MA owing to both the greater apparent relative density and the larger specific surface area of CNTs.

Being different from the stable modification effect of Gr, at relatively low CNTs dosages, *CII* of CNTs-MA underwent marginal increment, which also attributed to the CNTs agglomeration. Nevertheless, the thermal conductivity and the thermal diffusivity of CNTs-MA followed favorable linear relationships with CNTs content, even though there was comparatively smaller area of contact surface between CNTs and asphalt that would attenuate the thermal conduction. This phenomenon indicated that the contact resistance between modifier particles also restrained the enhancement on thermal properties of asphalt binders. 

## 5. Conclusions

This experimental study analyzed the influence of Gr and CNTs on the thermal, physical and rheological properties of asphalt binders to comprehensively evaluate their utilization potentials as thermal conductive modifiers in solar harvesting and snow melting pavements. Thermal parameters tests, penetration tests, softening point tests, ductility tests, Brookfield viscosity tests and dynamic shear rheometer tests were applied to Gr-MA and CNTs-MA, in which the modifier contents were chosen as 1%, 2%, 3%, 4% and 5% by volume of matrix asphalt. FTIR characterizations and TLC-FID tests were carried out to seek the modification mechanism and explore the influence factors on the modification effects. The following conclusions were drawn.

Gr and CNTs linearly increased the thermal conductivity and thermal diffusivity of asphalt binders with the increase of modifier content. Gr was more effective than CNTs. 5% Gr resulted in over 300% (3 times) increments in the thermal conductivity and thermal diffusivity of asphalt binders at 20 °C, and 5% CNTs only gave rise to approximately 72% (0.7 times) increments in the two parameters. Meanwhile, Gr and CNTs decreased the volumetric specific heat capacity of asphalt binders at 20 °C. The use of Gr resulted in a greater reduction. Increasing the interface thermal resistance between modifiers and asphalt as well as the contact resistance between modifier particles, the larger specific surface area of CNTs, compared with Gr, mitigated the real effects of thermal conduction modification.

Gr and CNTs promoted the consistency, high-temperature stability, apparent viscosity, stiffness, elasticity, and rutting resistance of asphalt binders, and reduced their temperature susceptibility (TS). All these features were conductive to enhancing the high-temperature properties of asphalt binders. In comparison, CNTs-MA gained more significant enhancement than Gr-MA on situation of high temperatures and high modifier contents. Additionally, regarding TS reduction, whereas CNTs was more effective at high temperatures, Gr performed better at moderate temperatures.

CNTs reduced the low-temperature performance of asphalt binders, including cracking resistance and ductility, as modifier content increased in a broad temperature range. However, although Gr weakened asphalt binder’ resistance to cracking at the temperatures between −10 °C and 30 °C based on the results of ductility tests and the low-to-intermediate temperature sweeping of DSR tests, it could eventually reduce the stiffness of asphalt binders at extremely low temperature according to the complex shear modulus master curves, possibly due to its unique crystal structure.

In this study, 3% Gr by volume of matrix asphalt performed with the best potential for the application of solar harvesting and snow melting pavements. Being calculated from the results of the linear regressions, the thermal conductivity, thermal diffusivity, and volumetric specific heat capacity of 3% Gr-MA could reach 0.4153 W/mK, 0.2533 mm^2^/s and 1.6398 MJ/m^3^ K at 20 °C, respectively. The enhancement of thermal properties can increase the work efficiency of solar harvesting and snow melting pavements, decrease the pavement surface temperature and further alleviate the urban heat island (UHI) effect.

This article opens up new opportunities and possibilities for the use of Gr as a promising thermal conductive modifier in asphalt pavements. The conclusions were obtained from the samples with a particular type and size. Further research on Gr and CNTs with other types and sizes is also necessary for consolidating or even generalizing the conclusions of this study.

## Figures and Tables

**Table 1:** Properties of matrix asphalt.

Properties	Values
Penetration (25 °C)	81.9 dmm
Softening point	43.8 °C
Ductility (15 °C)	>1000 mm
Flashing point	345 °C
Solubility in trichloroethylene	99.8%
Relative density (15 °C, compared with water)	1.03

**Table 2:** Physical parameters of Gr.

Properties	Values
Color	Dark grey
Layers	1–3
Single-layer rate (%)	>80
Carbon content (%)	98
Water content (%)	<2
Particle size distribution D_50_ (μm)	7–12
Particle size distribution D_90_ (μm)	11–15
Specific surface area (mm^2^/g)	50–200
Bulk Density (g/cm^3^)	0.06–0.09
Apparent relative density (15 °C, compared with water)	1.692

**Table 3:** Physical parameters of CNTs.

Properties	Values
Color	Black
Purity (%)	>97%
Ash content (%)	<2.5%
Tube diameter (nm)	3–15
Tube length (μm)	15–30
Specific surface area (mm^2^/g)	250–270
Bulk density (g/cm^3^)	0.01–0.02
Apparent relative density (15 °C, compared with water)	1.870

**Table 4:** The performance grade (PG) of Gr-MA and CNTs-MA.

Modifier Content (%)	PG (High Temperature Part)	
	Gr-MA	CNTs-MA
0	64	64
1	64	64
2	64	64
3	64	70
4	70	82 ^1^
5	70	82 ^2^

^1,2^ Estimated by the variation tendency of rutting factor.

**Table 5:** SARA fraction of Gr-MA.

Gr Content (%)	The Percentage of Components/%				Collide Instability Index *CII*
	Saturate	Aromatic	Resin	Asphaltene	
0	24.5133	35.9618	32.2616	7.2632	0.4658
1	24.7825	36.0740	32.0190	7.1246	0.4686
2	24.7520	36.6307	31.3259	7.2914	0.4715
3	25.3036	35.7349	31.7220	7.2395	0.4824
4	25.6996	36.6717	30.2614	7.3674	0.4940
5	25.5832	35.9562	30.1862	8.2743	0.5119

**Table 6:** SARA fraction of CNTs-MA.

CNTs Content (%)	The Percentage of Components/%				Collide Instability Index *CII*
	Saturate	Aromatic	Resin	Asphaltene	
0	24.5133	35.9618	32.2616	7.2632	0.4658
1	24.4804	36.0184	32.1995	7.3018	0.4659
2	24.4294	36.0929	32.1173	7.3603	0.4661
3	24.8536	35.7316	29.2486	10.1663	0.5389
4	25.7042	36.0467	26.6996	11.5494	0.5937
5	25.6311	37.4823	24.2933	12.5934	0.6188

## Data Availability

The data presented in this study are available on request from the corresponding author.
